# Interest rate volatility and financing of Islamic banks

**DOI:** 10.1371/journal.pone.0268906

**Published:** 2022-07-26

**Authors:** Muhammad Nouman, Maria Hashim, Vanina Adoriana Trifan, Adina Eleonora Spinu, Muhammad Fahad Siddiqi, Farman Ullah Khan

**Affiliations:** 1 Institute of Business and Management Sciences (IBMS), The University of Agriculture Peshawar, Pakistan; 2 Department of Economic Disciplines, Aurel Vlaicu University of Arad, Arad, Romania; 3 School of Management, Xi’an Jiaotong University, Xi’an, China; University of Almería, SPAIN

## Abstract

Despite a direct ban on charging interest, interest-based benchmarks are used as a pricing reference by a majority of Islamic banks, due in part to the absence of stable and widely- published alternatives. Benchmarking interest rate exposes Islamic banks to the problems of conventional banks, particularly the interest rate risk. Against this backdrop, the present study empirically examines the dynamic linkage between the interest rate volatility and the financing of Islamic banks. The empirical analysis is carried using evidence from the Islamic banking industry of Pakistan during the time period 2006–2020. The multivariate Johansen and Jusiles Co-integration test and Vector Error Correction Model (VECM) are used as the baseline econometric models. Moreover, the DCC-GARCH model is employed for robustness and ensuring the consistency of results. The results indicate that a significant long-term and short-term relationship exists between the interest rate volatility and the financing of Islamic banking industry providing significant evidence for co-movements and convergence. These findings suggest that paradoxical as it may seem, the financing of Islamic banks operating within a dual banking system is subject to interest rate risk, mainly due to benchmarking interest rate, which in-turn makes Islamic banks vulnerable to the rate of return risk and withdrawal risk. Moreover, corporate financing, in particular, is more vulnerable to interest rate risk.

## Introduction

Islam is a complete code of life [1]. In the Islamic society, the framework for all activities is provided by Islamic rules and laws known as *Shari’ah* [2, 3]. It governs all aspects of Islamic matters including faith, worship, social, cultural, political and economic concerns of Islamic societies [2–4]. *Shari’ah* also permeates financial transactions within the Islamic economic system and provides basic tenets for a unique Islamic financial system to ensure wellbeing of the overall society [1]. The Prohibition of *Riba* (lit. addition), i.e., interest in general lies at the core of Islamic finance system [5, 6].

*Riba* or interest is basically an increase or extra amount [7] that is paid by the lender as a premium [8]. It signifies the return on such transactions which involves the exchange of money for money and the payment of certain or agreed amount of money in case of any delay in payment of loan. The *Shari’ah* has forbidden *Riba* because it is harmful and vicious [7, 9], as it generates economic imbalances [8, 10, 11] and exploitations [12]. Thus, as per *Shari’ah* doctrine and the Islamic Finance philosophy, lender will lend money without expecting any return.

From Anas ibn Malik (Gbpwh): “The Prophet said: ‘When one of you grants a loan and the borrower offers him a dish, he should not accept it; and if the borrower offers a ride on an animal, he should not ride, unless the two of them have been previously accustomed to exchanging such favours mutually’.” (See Baihaqi, 1344 H, Kitab al-Buyu’, Bab kulli qardin jarra manfa‘atan fa huwa riban)

Prohibition of interest is the essence of Islamic financial system. However, simply describing the Islamic financial system as merely interest-free does not show a true representation of the system [2]. Besides prohibition of interest, it is also characterized by other principles including protection of property rights, individuals’ duties and rights, risk sharing and promotion of entrepreneurship, prohibition of speculative behavior, and the sanctity of contractual obligations [2, 13, 14].The Prohibition of various economic ills in the *Shariah* including interest, gambling, and excessive risk are based on the principle of providing justified ground to all parties involved in the transaction and safeguarding the interest and benefits of all stakeholder [8, 15–18]. Consequently, Islamic commercial banking is conceived as a value based banking system which should be based on principles of Islamic ethics, values, social and Islamic legal norms.

Islamic banking, being underpinned by Islamic finance principles, involves risks, balance sheets structures, and operations that are by far different from their conventional counterparts (IMF, 2017). Moreover, it is based on a unique set of *Shariah* compliant asset based financial products which makes it distinctive from the interest based conventional banking [3, 19]. However, the contemporary regulatory framework that governs Islamic banking, the dual banking environment within which the Islamic banks operate, and the current business model of Islamic banks posse different challenges to Islamic banks [20], particularly in terms of maintaining their unique identity and independence.

Amongst these challenges is the issue of benchmarking Islamic banking products to interest rate (Ahmad, 2018). That is, though Islamic banks do not charge interest; interest-based benchmarks are used as a pricing reference by a majority of Islamic banks, due in part to the absence of independent, stable and widely-published alternatives [21–25]. Presently, Islamic banks are using different interest-based benchmark rates for the determination of its cost of funds, and consequently the prices of its financing products e.g., London Interbank Offered Rate (LIBOR), Kuala Lumpur Interbank Offered Rate (KLIBOR), Karachi Interbank Offered Rate (KIBOR), and Cost of funds index (COFI) etc. [26, 27].

It is generally claimed that Benchmarking interest rate in Islamic banking does not violate the *Shariah* rulings The advocates of Islamic banking and finance are of the view that benchmarking interest for determining the profit of any permissible transaction does not render the transaction as invalid or *Haram*. Rather, it is the nature and the mechanism of the transaction that determines its validity or otherwise [22]. However, it is not without implications [28]. Benchmarking interest rate leads to invariably strong links between the Islamic and the conventional banking systems [26, 29]. Moreover, it exposes Islamic banks to the problems of conventional banks, particularly the interest rate risk [25, 30–32]. The interest rate risk refers to the effect of unanticipated changes in interest rate on the cash flows, profits, and/or net worth of a bank [33, 34]. This risk arises through mismatch in the liabilities, assets’ maturity, and off balance sheet positions due to fluctuations in the interest rate because the present value of banks’ future cash flows, and in certain cases the cash flow itself, changes with fluctuations in the interest rate [35]. This ultimately results in the volatility of bank’s income and net worth [36, 37].

Building upon these notions, several studies conducted around the world have empirically investigated the impact of benchmarking interest on different aspects of Islamic banks [See for example, 25, 31, 36, 38–41]. However, an important concentration apparent within the extant literature is the overwhelming attention given to the effect of benchmarking interest rate on the deposits, profitability, risk profile, and stock prices of Islamic banks; whereas the financing side of Islamic banks remains relatively unexplored. To bridge this gap the present study empirically investigates the effect of benchmarking interest rate on the financing of Islamic banks. Hence, this paper contributes to the extant literature in many ways. First, it provides empirical evidence regarding the vulnerability of Islamic banks to the interest rate risk. Second, it conceptualizes the implications of benchmarking interest rate and how it exposes Islamic to the rate of return risk, withdrawal risk, and the displaced commercial risk. Finally, it implies rich agenda for policy and practice.

The research on the benchmarking interest and its implications is also timely and desirable keeping in-view the changing dynamics in the international and local financial environment. Interestingly, after 60 years from the date of its inception, the LIBOR has finally been phased out by the end of 2021 [42, 43]. Effective 31th December 2021, LIBOR has been abandoned for pricing new loans in US. It has been replaced with Secured Overnight Financing Rate (SOFR). This is because it has been criticized for being subjective and prone to manipulation by banks. Similarly, the equivalent benchmark rates (like Euribor) are also losing their attraction. To grab their place alternative benchmark rate like British SONIA (Sterling Overnight Index Average), Swiss SARON (Swiss Average Rate Overnight) and US SOFR (Secured Overnight Funding Rate) are already being experimented with [44]. Similarly, the prevailing Islamic interbank benchmark rate (IIBR) has also failed to prove as independent reference rates due to its strong linkage with the conventional benchmark rates [26, 31, 33, 44–47]. Therefore, a strong need is felt for an independent Islamic benchmark rate that does not face the lacunae that LIBOR and IIBR face.

The reset of the paper is organized as follows: section 2 presents review of the literature followed by the research methodology in section 3. Empirical results and findings are presented and contextually discussed in section 4, while section 5 concludes the paper and presents policy implications.

## Literature review

Islamic economic system is largely based on some certain encouragements and prohibitions [22, 48]. In Islamic economic system, *Riba* (Interest) is prohibited whereas trade is permitted [8, 12, 22]. “Allah has allowed (profit from) trade and prohibited *Riba*” (verse 2: 275 of the Holy Qur’an). Such teachings of *Qur’an* imply that financial activities involved in an Islamic economic system should be asset-backed transactions and businesses. This suggests that all the financial transactions ought to illustrate real transactions or sale of goods, benefits, or services [22].

Islamic banking, being based on Islamic principles, discourages interest (*Riba*) and endorses the concept of profit sharing [9, 49]. Therefore, it is based on a range of distinctive *Shariah* compliant financial products [3, 19]. Contrary to the conventional banking, whose core business is interest based borrowing and lending, the practice of Islamic banking is fundamentally based on various types of participatory and non-participatory contracts devised from Islamic Law [50, 51].

Participation, also called profit and loss sharing, establishes a paradigm that is believed to represent an ideal Islamic finance system [22, 24, 52–54]. It allows a financial institution to earn profit on invested capital if the financial institution is willing to accept loss in case of the project failure [55, 56]. Modes of participatory financing include *Mudarabah* (partnership of capital) and *Mudarabah* and *Musharakah* (partnership of capital and skill). The non-participatory modes on the contrary do not involve profit and loss sharing and usually entail a rather predetermined return [19, 57]. These modes include *Murabahah* (‘mark-up’ or cost plus sale), *Ijarah* (lease), *Salam* (differed delivery), *Istisna’* (commission to manufacture), *Bai Muajjal* (deferred payment), *Jo’alah* (service fee), and *Qard Al Hasana* (charity loan) [58, 59].

Though, Islamic banking is grounded on the principle of avoiding interest. However, paradoxically the argument of Islamic banking being completely free and independent of interest rate is dubious at best [29]. In reality, the interest rates have deep roots in the present edifice, where Islamic financial institutions do not have independent mechanisms for pricing. By far, Islamic banks operate in a dual banking system throughout the world (excluding Iran and Sudan where the whole financial system has been Islamized). Thus, to cope with the sever competition the Islamic banks, operating within dual banking system, are consistently mimicking and matching their prices with their interest based conventional counterparts [20, 29].

For the effective pricing of Islamic banking products and decoupling of their rate from that of their conventional counterparts, Islamic banks have made efforts to create their own benchmark rate [46]. Consequently, Islamic banks came up with an Islamic benchmark rate called the Islamic interbank benchmark rate (IIBR). The IIBR was introduced by Thomson Reuter Corp in cooperation with a consortium of 19 Islamic banks form Gulf Cooperation Council and several well-known financial industry associations including Islamic Development Bank (IDB), the Statistical Economic and Social Research Center for Islamic Countries, the Hawkamah Institute for Corporate Governance, the Bahrain Association of Banks, the Association of Islamic Banking Institutions Malaysia, and Islamic regulatory body Accounting and Auditing Organization for Islamic Financial Institutions (AAOIFI).

However, this benchmark rate has several shortcomings including its limited applicability, the lack of tools that the central banks and commercial banks may use to adjust liquidity and significant linkage of its spread with that of the conventional interest based benchmark rate e.g. LIBOR [26, 31, 45, 46]. Thus, the efforts to develop an effective independent Islamic benchmark rate have failed [31, 47]. Consequently, interest-based benchmarks are used as a pricing reference by a majority of Islamic banks in major parts of the world [22, 24, 25].

Though benchmarking interest rate in Islamic banking does not violate the *Shariah* rulings; it is heavily criticized and has become a subject of debate in the Islamic banking and finance literature [28]. Many Islamic scholars criticize benchmarking interest as “not being sufficiently based on real economic activity, a key principle in Islamic finance” [23]. Moreover, it violates the basic philosophy of Islamic banking and finance [21] and makes the transactions of Islamic banks indistinguishable from those of conventional banks [22, 25].

Furthermore, it is accused of inducing interest rate risk in Islamic banks [25, 30–32, 37]. The interest rate in turn makes Islamic banks vulnerable to the substitution effect, particularly in the dual banking system where profit oriented customers can readily choose between Islamic and conventional banks which could lead to the changes in the deposit profile and an imbalance in the deposits [34, 60]. To avoid this problem, Islamic banks would have to pay higher return to depositors [33]. However, this could lead to the problem of shrinkage in the profit margin of Islamic banks because bank may not be able to increase the earnings from the assets to keep pace with the interest rate movements [34, 36].

On the contrary, few advocates of Islamic banking favor benchmarking interest rate [See for example 7, 24, 28, 61]. They justify Interest as a benchmark on the grounds that Islamic banking, being a niche market, has to co-exist with the conventional banking (See for example, Hamoud, 1994, Usmani, 2002a). Islamic banks have to use interest rate as a benchmark to remain competitive and be able to attract deposits from customers [28]. Moreover, it helps in avoiding arbitrage in the dual banking environment [28, 62]. Shariah adviser Aznan Hasan an advisor to Malaysia’s stock exchange Bursa Malaysia said in an interview:

*If you were to have in one country, two benchmarks—Islamic and conventional—together, it won’t be easy for a country to adopt the situation. People will arbitrage. Once they see conventional financing is much better, they will go for conventional. Once they see Islamic is much better, they will go for Islamic. In that situation, it will give a big turbulence to a country*.

This ongoing debate has opened new avenues for research. Several studies have investigated the effect of interest rate on different aspects of Islamic banking. For example, various studies have considered the impact of interest rate on the profitability of Islamic banks [See for example 63–65]. Similarly, many researchers have investigated the effect of interest rate on deposits of Islamic banks [See for example 36, 38–40, 60, 66–69]. Whereas, the impact of interest rate on the stock prices of the Islamic banks was considered by Ajmi, Hammoudeh [70], Aloui, Hammoudeh [71], Ayub and Masih [72], Fakhfekh, Hachicha [73], Hussin, Muhammad [74], and Shamsuddin [41].

On the contrary, the literature on the link between the interest rate and the financing of Islamic banks is relatively limited. Few studies have investigated the effect of interest rate on the demand and supply of Islamic financing in the Malaysian context. For example, Yusoff, Rahman [75] attempted to investigate the effect of interest rate on the supply of Islamic financing in Malaysia. While Yap and Kader [60] and Kader and Leong [32] empirically investigated how interest rate fluctuations in a dual banking system affect the demand for the financing of Malaysian Islamic banks. However, by far, empirical evidence on the linkage between interest rate volatility and financing of Islamic banks and its consequent implications for Islamic banks remains relatively scarce. Therefore, the present study empirically investigates the dynamic linkage between interest rate and the financing of Islamic banks. For this purpose we propose the following hypothesis:

***Hypothesis 1*:** *There is a significant dynamic linkage between interest rate volatility and the amount of financing extended by Islamic banks*.

## Research methodology

### Data and variables

In this research secondary data has been used. Weekly time series data on the financing mix of the overall Islamic banking industry for the period March-2006 to March-2020, having 734 observations, was obtained from the official website of State Bank of Pakistan (SBP) and Islamic banking bulletins issued by the Islamic banking department of SBP. The financing mix of the overall Islamic banking industry was considered which includes the financing of all scheduled Islamic banks operating in Pakistan. Since Islamic banks are offering financing to different sectors including corporations, SMEs, agriculture, consumer financing, commodity operations, and several other sectors. Therefore, overall funding extended to different sectors are considered as the variables of the study including: i) corporate financing, ii) SMEs financing, iii) agriculture financing, iv) consumer financing, v) commodity operations financing, vi) financing to other sectors, and vii) Total financing. Similarly, the time series data of the interest rate was also collected from the SBP official website. Since Islamic banks in Pakistan use Karachi Inter-Bank Offer Rate (KIBOR) as a benchmark for determining the cost of financing. Therefore, lagged KIBOR was used as proxy for the interest rate. The lagged KIBOR was used because changes in the KIBOR are not expected to be concurrently reflected in the financing of Islamic banks.

### Baseline tests and model

Different time series models were applied for the analyses of the data using the Eviews application. Conventional time series models including the tests for Unit root, Cointegration Test and test for Error correction were applied as the baseline tests. Application of these procedures is consistent with the norms of time series analysis and similar studies in the Islamic banking research [See for example: 31, 76–78].

The baseline tests were applied in three phases. In the first phase the Augmented Dicky-Fuller (ADF) test [79] and Phillip-Perron (PP) test [80] were used for testing the unit root problem at level and 1^st^ difference of each series. The equation for ADF test is given as follows:

$$\Delta Z_{t}=\gamma_{1}+\beta z_{t-1}+\pi \sum_{t=1}^{k}{\Delta Z}_{t-1}+\mu_{t}$$ (1)

In above equations Δ is the 1^st^ difference operator, *γ*_1_ is the constant, *μ*_1_ is the error term, and the coefficient *β* = (*ρ*−1). For above equations the null hypothesis is H_0_: *ρ* = 1 or *β* = 0, which means that the time series are non-stationary i.e., having a unit root. The alternative hypothesis is H_1_: *β* < 0, that is, the time series is stationary and does not have the unit root problem. For optimal lag selection the Akaike information criterion (AIC), Schwarz information criterion (SIC), and Hannan-Quinn criterion (HQC) are used.

On the other hand, for Phillip-Perron (PP) test the following equation is used:

$$\Delta z_{t}=\alpha_{1}+\pi z_{t-1}+\mu_{t}$$ (2)

Where, *α*_1_ is the constant term, and *μ*_1_ is the error term. Both ADF and PP test produce the same results, however, they differ mainly on how the heteroskedasticity and serial correlation in the error terms are dealt with [81]. In the present study, both PP test and ADF test are applied for cross-checking and ensuring the accuracy of the results.

In the second phase, the unit root tests were followed by the multivariate Johansen and Jusiles (JJ) Co-integration test [82] for testing long-run association among interest rate volatility and the financing of Islamic banking industry. The general form of JJ model is given as follows:

$$\Delta x_{t}=\phi x_{t-1}+\sum_{t=1}^{k-1}\vartheta_{i}{\Delta x}_{t-i}+{\lambda y}_{t}+\varepsilon_{t}$$ (3)

Where, *x*_*t*_ is (nx1) vector of *I*(1) time series variables, *λy*_*t*_ is a vector of constants, *ϕ* is the (n × n) matrix of long term parameters of the error correction, while *ϑ*_*i*_ represents the (n × n) matrices of short term parameters of lagged difference factor.

The JJ Cointegration was estimated using a three-step procedure: In the first step the order of integration was determined for each variable using the unit root tests. In the second step the optimal lag length was selected using the basic criterion including Akaike information criterion (AIC), Schwarz information criterion (SIC), and Hannan-Quinn criterion (HQC). Whereas, in the last step the cointegrating vectors were determined using two tests namely Trace test, and Maximum Eigenvalue test, which eventually indicate the presence or absence of cointegration. The formulas for Trace test, and Maximum Eigenvalue test respectively are given as follows:

$$\lambda_{trace(r)}=-T\sum_{i=r+1}^{k}In(1-{\hat{\lambda }}_{i})$$ (4)

$$\lambda_{max(r,r+1)}=-TIn(1-{\hat{\lambda }}_{r+1})$$ (5)

Where r represents the number of cointegrating vectors under the null hypothesis while ${\hat{\lambda }}_{i}$ are the estimated Eigen values from the estimated matrix *ϕ*. After determining the cointegration, Vector Error Correction Model (VECM) was applied in the third phase to examine speed of the adjustment process towards the long-term equilibrium i.e., how errors are reconciled by time series in pursuing long run equilibrium path. The mathematical presentation of VECM is as follows:

$$\Delta y_{t}=k+\sum_{i=0}^{k}{\beta 1}_{i}{\Delta X1}_{t-i}+\sum_{i=0}^{k}{\beta 2}_{i}{\Delta X2}_{t-i}\ldots +\sum_{i=0}^{k}{\beta n}_{i}\Delta {Xn}_{t-i}+{Z_{1}EC1}_{i-1}+\varepsilon_{1t}$$ (6)

Where Δ is the 1^st^ difference operator, Y_*t*_ is the dependent variable, *K* is constant, X1….Xn are the independent *I*(1) variables, *β*1 to *βn* represent the coefficient of independent variables X1…. Xn respectively, *EC1*_*t* -1_ is the error correction term, Z_1_ is the coefficient of error correction term, and *ε*_1*t*_ is the residual or error term.

### Robustness test

Besides the conventional time series models, the present study also employs the Dynamic Conditional Covariance (DCC)-Generalized Autoregressive Conditional Heteroscedasticity (GARCH) model for robustness which was proposed by Engle [83]. One of the limitations of the conventional time series models is that these operate under the assumption of constant variance [84]. To resolve this issue Engle [85] introduced the Autoregressive Conditional Heteroskedastic (ARCH) process to allow for changes in the conditional variance over time as the function of past errors. Consequently, the unconditional variance becomes constant. However, this model calls for a relatively long lag in the conditional variance equation. Moreover, it imposes a fix lag structure to shun the issues with negative variance parameter estimates [85, 86].

To resolve these limitations Bollerslev [84] introduced the Generalized Autoregressive Conditional Heteroskedastic (GARCH) process which is an extension of the ARCH class of models and allows for both a greater flexibility in the lag structure and a longer memory. Afterwards, Bollerslev [87] proposed a multivariate conditional heterskedatic model with time varying conditional covariances and varainces, but constant conditional correlations. This model was termed as CCC-GARCH (constant conditional correlations GARCH). The underlying assumption of the time varying conditional covariances but constant conditional correlations simplifies the procedures of estimation and inference by a greater extant and also allows for the comparisons between different periods [87]. However, the assumption of the time invariant conditional correlations of CCC-GARCH is far restrictive in practice [88]. Therefore, many authors have proposed alternative models with time-varying conditional correlations. Engle [83] proposed a generalization of CCC-GARCH called the Dynamic Conditional Correlation (DCC)-GARCH wherein the conditional correlation matrix is time-dependent. Thus, it is an extension of CCC-GARCH model with time-varying conditional correlations.

The DC-GARCH models offer a class of flexible non-linear multivariate models that can be used for analyzing and forecasting volatility of multiple time series and their correlations, particularly when the underlying volatility varies over time [89]. Coupled with the flexibility of the univariate GARCH models, the DCC-GARCH offers parsimonious parametric models for the estimation of correlations. That is, the multivariate DCC-GARCH has inherent flexibility which helps in simplifying the problem of multivariate conditional variance estimation. This is because the DCC model allows for two stage estimations, where the first stage involves the estimation of the univariate GARCH models for reach residual series, while the second stage involves the use of residuals estimated in the first stage (i.e., the residuals transformed by their standard deviations) for the estimation of the dynamic correlation parameters.

Suppose *y*_*t*_ = [*y*_1*t*_, *y*_2*t*_]′ is a 2x1 vector of the data series. The conditional means equations can be presented as the following reduced-form VAR:

$$A(L)y_{t}=\varepsilon_{t}\;\varepsilon_{t}∼N(0,H_{t})\;where\;t=1,2,\ldots T$$ (7)

Where *A*(*L*) presents a matrix in the lag operator *L*, and *ε*_*t*_ = [*ε*_1*t*_, *ε*_2*t*_]′ is the vector of innovations with the following variance-covariance matrix:

$$H_{t}=D_{t}R_{t}D_{t}$$ (8)

Where *H*_*t*_ represents the conditional variance matrix, *R*_*t*_ represents the time varying correlational matrix containing conditional correlations, while *D*_*t*_ is a n x n diagonal matrix of time varying standard deviations form univariate GARCH models having $\sqrt{h_{it}}$ on the diagonals.


$$D_{t}=\left[\begin{array}{cccc} \sqrt{h_{1t}} & 0 & \cdots & 0\\ 0 & \sqrt{h_{2t}} & \ddots & \vdots \\ \vdots & \ddots & \ddots & 0\\ 0 & \cdots & 0 & \sqrt{h_{nt}} \end{array}\right]$$

The univariate GARCH (X, Y) model is used to estimate the conditional variance (*h*_*it*_) for different investment portfolios as indicated in the following equation:

$$h_{it}=\omega_{i}\sum_{p=1}^{P_{i}}\alpha_{ip}\;r_{it-p}^{2}+\sum_{q=1}^{Q_{i}}\beta_{iq}\;h_{it-q}$$ (9)

Where i = 1,2,…., n with the usual GARCH restrictions of stationarity and non-negativity being imposed such as $\sum_{p=1}^{P_{i}}\alpha_{ip}+\sum_{q=1}^{Q_{i}}\beta_{iq}$ < 1 and non-negativity of variances. The subscript on the individual *P* and *Q* for each series indicates the lag lengths chosen which do not need to be necessarily the same. The specification of the univariate GARCH models is not limited to merely to the standard GARCH (p,q). Rather it can include any GARCH process having normally distributed errors that fulfills the appropriate non-negativity constraints and the stationarity conditions [90]. The proposed dynamic correlation structure for the DCC(M,N)-GARCH model can be given as:

$$Q_{t}=\left(1-\sum_{m=1}^{M}\alpha_{m}-\sum_{n=1}^{N}\beta_{n}\right)\bar{Q}+\sum_{m=1}^{M}\alpha_{m}(\epsilon_{t-m}\epsilon_{t-m}^{\prime })+\sum_{n=1}^{N}\beta_{n}\;Q_{t-n}$$ (10)

Where, $\bar{Q}$ represent the unconditional covariance of the standardized residuals derived from the 1^st^ stage estimation (i.e., univariate GARCH model). Thus, the *R*_*t*_ (in *Eq* 8) can be decomposed into:

$${R_{t}=Q}_{t}^{*-1}Q_{t}Q_{t}^{*-1}$$

Where,

$$Q_{t}^{*}=\left[\begin{array}{cccc} \sqrt{q_{11}} & 0 & \cdots & 0\\ 0 & \sqrt{q_{22}} & \ddots & \vdots \\ \vdots & \ddots & \ddots & 0\\ 0 & \cdots & 0 & \sqrt{q_{nn}} \end{array}\right]$$

So, $Q_{t}^{*}$ is the n x n diagonal matrix comprised of the square root of the diagonal elements of *Q*_*t*_. We primarily focus the conditional correlations in *R*_*t*_. Thus, unlike constant conditional correlation (CCC)-GARCH, the DCC-GARCH does not suffer with the problem of constant correlations. Similarly, unlike multivariate GARCH (M-GARCH), it does not have the difficulty of dimensionality. This is because the DCC-GARCH eases the assumption of constant correlation and enables time-varying correlations that can be measured with respect to the variables’ past values.

## Results and discussion

### Summary statistics

Table 1 presents annual averages for the study variables. The annual averages suggest that the corporate financing constitute the major portion (around 70%) of Islamic banks’ financing followed by commodity sector which represents financing extended to government for commodity operations financing. On the other hand, agriculture sector remains the least attractive sector for the Islamic banking industry followed by SMEs sector. These results are consistent with the findings of Nouman, Ullah [19] who also report similar results.

**Table 1:** Annual averages (average annual financing of overall industry in billion rupees).

Year	KIBOR * (%)	Total Financing	Corporate Financing	SMEs Financing	Agriculture Financing	Consumer Financing	Commodity Financing	Others **
2006	8.30	59.45	38.29	5.83	0.00	13.26	0.65	1.43
2007	8.78	86.95	56.00	8.52	0.00	19.39	0.96	2.09
2008	10.64	136.93	88.18	13.42	0.00	30.53	1.51	3.29
2009	11.88	145.25	94.70	11.18	0.00	29.05	6.97	3.34
2010	11.82	168.96	116.70	9.81	0.00	26.89	10.99	4.57
2011	12.56	191.38	140.04	9.96	0.19	26.63	11.30	3.26
2012	10.56	217.40	156.88	10.37	0.22	31.08	14.56	4.29
2013	8.85	284.17	202.12	12.65	0.28	36.06	27.62	5.44
2014	9.56	358.37	271.69	14.44	0.93	44.99	19.58	6.73
2015	6.82	527.08	398.03	15.87	3.22	58.47	33.31	18.18
2016	5.79	711.83	551.45	21.52	5.36	78.30	40.59	14.60
2017	5.72	1035.24	743.79	32.49	5.16	107.23	123.79	22.78
2018	7.01	1371.08	1007.25	43.78	4.44	141.13	156.01	18.47
2019	11.82	1558.71	1154.89	54.96	6.62	158.25	162.92	21.06
2020	10.47	1635.63	1246.74	49.02	6.54	161.77	150.33	21.24

* 1 week KIBOR rate

** Including Staff financing

### Baseline cointegration and VECM results

The Augmented Dicky-Fuller (ADF) test [79] and Phillip-Perron (PP) test [80] are used for testing the data stationarity. Table 2 presents results of Augmented Dickey and Fuller (ADF) test and Philips and Perron (PP) test at level and first difference. Results of both tests indicate that all variables have unit root problem at level. However, these variable become stationary at the first difference indicating that all variables are integrated at order 1 *I*(1). Hence, the Johansen and Juselius (JJ) co-integration test can be applied to check the long run association among the variables under consideration.

**Table 2:** Results of unit root test.

Variables	Augmented Dicky Fuller (ADF) test				Phillips—Perron (PP) test			
	At level		At first difference		At level		At first difference	
	*τ*	Pro.	*τ*	Pro.	*τ*	Pro.	*τ*	Pro.
Corporate Financing	-3.038443	0.1353	-5.957237	0.0001	-3.04617	0.1333	-25.4406	0.0000
SMEs Financing	-1.379737	0.8500	-3.768033	0.0314	-0.72603	0.9638	-6.59459	0.0000
Agriculture Financing	-1.010998	0.9307	-6.595182	0.0000	-3.91156	0.9102	-6.60906	0.0000
Consumer Financing	0.855359	0.9997	-6.154707	0.0000	0.716264	0.9995	-6.20161	0.0000
Commodity Financing	-2.126865	0.5120	-8.159308	0.0000	-1.83400	0.6671	-10.4342	0.0000
Other sectors Financing	-2.723863	0.2332	-13.13378	0.0000	-5.21445	0.2332	-12.198	0.0000
Total financing	-0.966195	0.9360	-5.118414	0.0011	-1.17317	0.9007	-5.08033	0.0012
Interest Rate	-2.147901	0.5051	-4.29417	0.0077	-1.63501	0.7622	-3.96501	0.0177

Table 3 present the results of Johansen and Juselius (JJ) co-integration test. The JJ co-integration is applied to evaluate the long run relationship among interest rate and the financing of Islamic banking industry. The Trace statistics and the Maximum-Eigen values indicate that there are at most seven (07) co-integrating equation, indicating that the variables are co-integrated. Consequently, based on the results it is concluded that long-run relationship exists between interest rate and the financing of Islamic banking industry. These results are consistent with the findings of Kader and Leong [32], Khalidin and Masbar [91], Yap and Kader [60], and Yusoff, Rahman [75].

**Table 3:** Results of multivariate Johansen and Juselius (JJ) cointegration test.

Hypothesized No. of CE(s)	Trace Statistic	Critical Value	Prob.**	Max-Eigen Statistic	Critical Value	Prob.**
None *	629.2249	197.3709	0.0001	179.2253	58.43354	0.0000
At most 1 *	449.9996	159.5297	0.0000	123.1880	52.36261	0.0000
At most 2 *	326.8116	125.6154	0.0000	111.1018	46.23142	0.0000
At most 3 *	215.7098	95.75366	0.0000	94.56068	40.07757	0.0000
At most 4 *	121.1491	69.81889	0.0000	43.69849	33.87687	0.0025
At most 5 *	77.45065	47.85613	0.0000	36.91135	27.58434	0.0024
At most 6 *	40.53929	29.79707	0.0020	28.82633	21.13162	0.0034
At most 7	11.71296	15.49471	0.1712	8.993764	14.26460	0.2867

Since, the financing of Islamic banks, extended to different sectors, and interest rate reveal cointegrating (long-run) relationships, VECM was assessed to model short-run dynamics of each scheme. Results from the VECM model are shown in Table 4. The results show that, the Error Correction Terms (ECTs) are negative and statistically significant for most of the equations indicating the propensity to adjust to any aberrations in the long-run equilibrium. The significance of these ECTs provides further proof for a cointegrating relationship among the interest rate and the financing of Islamic banks.

**Table 4:** Results of Vector Error Correction Model.

Error Correction:	D(IR-VOL)	D(CORPOR)	D(SMES)	D(AGRI)	D(CONSU)	D(COMM)	D(OTHER)	D(TOTAL)
CointEq1	-0.672253	-3.382036	-0.581539	0.430316	-24.46044	-2.560423	2.655683	-1.745189
	(0.11804)	(2.11383)	(0.77197)	(1.14907)	(35.6962)	(8.24812)	(2.38265)	(1.52503)
	[-5.69530]	[-1.59995]	[-0.75332]	[0.37449]	[-0.68524]	[-0.31043]	[1.11459]	[-1.14437]
CointEq2	-0.037377	0.043735	0.031475	0.493875	2.432885	0.506982	0.237529	0.127325
	(0.01292)	(0.23134)	(0.08449)	(0.12576)	(3.90666)	(0.90269)	(0.26076)	(0.16690)
	[-2.89339]	[0.18905]	[0.37255]	[3.92726]	[0.62275]	[0.56164]	[0.91091]	[0.76287]
CointEq3	0.318315	-2.442175	-0.420135	-0.206332	-26.88263	-1.974563	2.458692	0.171841
	(0.08369)	(1.49868)	(0.54731)	(0.81467)	(25.3082)	(5.84782)	(1.68927)	(1.08123)
	[3.80367]	[-1.62955]	[-0.76763]	[-0.25327]	[-1.06221]	[-0.33766]	[1.45548]	[0.15893]
CointEq4	0.050100	0.001641	-0.012469	-2.227717	-3.342971	-0.953983	-0.975892	-0.246477
	(0.05626)	(1.00753)	(0.36795)	(0.54769)	(17.0141)	(3.93136)	(1.13566)	(0.72688)
	[0.89049]	[0.00163]	[-0.03389]	[-4.06750]	[-0.19648]	[-0.24266]	[-0.85932]	[-0.33909]
CointEq5	-0.010013	-0.011126	0.009922	-0.027594	-0.513296	-0.067951	-0.017963	-0.017378
	(0.00151)	(0.02704)	(0.00988)	(0.01470)	(0.45668)	(0.10552)	(0.03048)	(0.01951)
	[-6.63032]	[-0.41143]	[1.00460]	[-1.87703]	[-1.12396]	[-0.64394]	[-0.58928]	[-0.89069]
CointEq6	0.027583	-0.072926	-0.017174	-0.423708	-3.977291	-0.905436	0.017069	0.027022
	(0.01477)	(0.26456)	(0.09662)	(0.14381)	(4.46758)	(1.03230)	(0.29820)	(0.19087)
	[1.86715]	[-0.27565]	[-0.17776]	[-2.94626]	[-0.89026]	[-0.87711]	[0.05724]	[0.14158]
CointEq7	-0.069444	0.708680	0.073959	0.316387	5.691700	-0.099243	-0.629024	-0.003684
	(0.02495)	(0.44684)	(0.16318)	(0.24290)	(7.54577)	(1.74356)	(0.50366)	(0.32237)
	[-2.78316]	[1.58598]	[0.45323]	[1.30254]	[0.75429]	[-0.05692]	[-1.24890]	[-0.01143]

The p-values for each co-integrating equation are presented in Table 5 to determine whether the short run relationship exists among variables. To calculate p-values the system equations have been used. The estimated coefficients of the ECT indicate the speed of adjustment towards the equilibrium point. The co-efficient of co-integrating Eq 1 indicates that 67.22% deviance in the benchmark rate from its equilibrium point recuperate in each period. The probability value of the error correction coefficient for the benchmark rate is significant (less than 0.05) and negative in sign, categorically proposing that short run causality exists, which moves from the benchmark rate to financing of Islamic banking industry in different sectors.

**Table 5:** Probability values for system equations.

	Coefficient	Std. Error	t-Statistic	Prob.
C(1)	-0.672253	0.118036	-5.695301	0.0001
C(2)	-0.037377	0.012918	-2.893386	0.0126
C(3)	0.318315	0.083687	3.803666	0.0022
C(4)	0.050100	0.056261	0.890491	0.3894
C(5)	-0.010013	0.001510	-6.630320	0.0000
C(6)	0.027583	0.014773	1.867147	0.0846
C(7)	-0.069444	0.024952	-2.783157	0.0155
C(8)	0.036347	0.125650	0.289269	0.7769
C(9)	-0.062175	0.091319	-0.680851	0.5079
C(10)	0.017809	0.012012	1.482567	0.1620
C(11)	-0.013637	0.013204	-1.032798	0.3205
C(12)	-0.152602	0.086830	-1.757486	0.1023
C(13)	-0.043072	0.038353	-1.123042	0.2817
C(14)	0.020206	0.032544	0.620872	0.5454
C(15)	0.013767	0.013713	1.003936	0.3337
C(16)	0.008078	0.001757	4.597213	0.0005
C(17)	0.003570	0.001527	2.338428	0.0360
C(18)	-0.026675	0.015059	-1.771422	0.0999
C(19)	-0.009682	0.009788	-0.989160	0.3406
C(20)	-0.019166	0.019070	-1.005032	0.3332
C(21)	0.007437	0.015362	0.484134	0.6363

The results of VECM reveal that seven equations are negative in sign and significant which indicates that the short run association exists between the time series i.e., they are capable to adjust their errors from long run equilibrium in a sufficient way. These findings provide strong evidence for co-movements and convergence. Therefore, Islamic banks, though operating on interest free ideologies, are prone to interest rate risk. Thus, our findings confirm the view of Archer and Karim [25], Bacha [34], and Rosly [30] who suggest that benchmarking interest rate exposes Islamic banks to the interest rate risk. This calls for the development of a unique *Shariah* compliant non-interest based benchmark for Islamic banks that is significantly decoupled from the conventional benchmarks and could be used as a robust indicator of the average expected cost of short term interbank market funding.

### Potential biases and robustness tests

The cointegration and VECM indicate the presence of long term co-movement between interest rate volatility and financing of Islamic banks, and the short term error correction respectively. However, these models are limited in terms of estimating the time variant volatility of the said time series and the dependence in their co-movements. Furthermore, these models do not indicate the pairwise linkage between interest rate and financing of Islamic banks in different sectors. Therefore, it is difficult to conceptualize which sectors are more vulnerable to the interest rate risk. To cover these limitations the multivariate Dynamic Conditional Covariance (DCC)-Generalized Autoregressive Conditional Heteroscedasticity (GARCH) model has been employed for the analysis of the data.

Unlike alternative methods, the DCC framework offers a flexible approach for the estimation of the multivariate GARCH models and allows to model separately the conditional correlations and conditional variances [92]. Thus, due to its inherent flexibility, the DCC-GARCH model provides accurate approximation of time-varying correlations and volatilities. Therefore, keeping in view its inherent flexibility and its ability to estimate the pairwise linkage between the variables, the DCC-GARCH seems as one of the most appropriate models for estimating the volatility based relationship between different streams of Islamic financing in Islamic banks and the interest rate and checking the robustness of the results.

#### Stationarity and residual diagnostic tests

The GARCH family has various assumptions including the presence of data stationarity, autocorrelation, and ARCH effect in the data. Furthermore, the conditional distribution in the GARCH models is supposed to follow a parametric distribution. To test these assumptions and justify the usage of DCC GRACH several preliminary tests have been performed including the ADF test, Ljung-Box Q-stat test, Lagrange multiplier (LM) test, ARCH effect test, and the Shapiro-Wilk test. The data was transformed using the first difference transformation to resolve the unit root problem. Table 6 reports the results of these preliminary tests.

**Table 6:** Preliminary tests for DCC-GARCH model.

Variables	ADF Test		Q-Statistics		LM Test		ARCH effect		Shapiro-Wilk	
		Prob.	Q-stat	Prob.	T	Prob.	Z	Prob.	Z	Prob.
Corporate Finan.	-5.957	0.000	81.566	0.000	91.799	0.000	441.19	0.000	1.153	0.123
SMEs Financing	-3.768	0.031	03.010	0.033	32.024	0.000	238.10	0.000	1.313	0.095
Agriculture Finan.	-6.595	0.000	24.091	0.000	24.151	0.000	140.37	0.000	0.949	0.171
Consumer Finan.	-6.154	0.000	36. 943	0.000	37.098	0.000	48.967	0.000	0.541	0.294
Commodity Finan.	-8.159	0.000	32.161	0.000	44.255	0.000	169.36	0.000	0.855	0.192
Other sectors Fin.	-13.133	0.000	13.402	0.045	13.083	0.001	207.793	0.000	0.917	0.163
Total financing	-5.118	0.001	33.251	0.000	52.215	0.000	154.321	0.000	0.361	0.358
Interest Rate	-4.294	0.007	05.261	0.000	41.65	0.000	214.24	0.000	0.052	0.479

The ADF test is used to check the presence of unit root or stationarity in the data. The underlying null hypothesis of this test is that the time series has unit root. The result of ADF test indicates that all variables are significant at 5% level of confidence, which denies the presence of unit root in the data, and thus the data satisfies the assumption of the stationary time series. Similarly, the Ljung-Box Q-stat test is applied to check the serial correlation/autocorrelation in the data. This test has the null hypothesis that time series variables do not have autocorrelation. Results show that null hypothesis is rejected for all the variables at 5% significance value which indicates that the time series have autocorrelation. The LM test also confirms the results of Q-statistics.

Similarly, the ARCH test is used to check heteroscedasticity or ARCH effect in the data. The results of this test highlight the presence of ARCH effect in the data. On the other hand, the Shapiro-Wilk test is used to assess the normality of distribution. The null hypothesis of this test is that the distribution is normally distributed. Since, probability value of each variable is insignificant at 5% level of significance. Therefore, we can conclude that all variables are normally distributed. Hence all preliminary conditions are satisfied for the DCC-GARCH Model.

#### Multivariate DCC-GARCH models results

The time varying volatility has important role in understanding the behavior of portfolio diversification among different streams of Islamic financing. Therefore, the DCC-GARCH model developed by Engle [83] is selected to address the time-varying volatilities and correlations among the selected streams of financing. However, the DCC-GARCH is predominantly based on the Gaussian distribution which might be inefficient for heavy-tailed distributions [93]. Therefore, along with the Gaussian-DCC GARCH model, we have also used the t- DCC GARCH variant of DCC-GARCH model for robustness purpose following the previous studies of Najeeb, Bacha [94], Jaffar, Dewandaru [95], Buriev, Dewandaru [96], Joyo and Lefen [97]. This variant assumes multivariate t-distribution and is thus suitable for heavy-tailed data [93, 97]. Table 7 reports the results of the Gaussian-DCC GARCH and t- DCC GARCH models.

**Table 7:** Maximum likelihood estimates of DCC-GARCH models.

Parameter	DCC-GARCH with t distribution *				Gaussian-DCC model **			
	*λ* 1	*λ* 2	Prob.	1- (*λ*_1_+ *λ*_2_)	*λ* 1	*λ* 2	Prob.	1 - (*λ*_1_+ *λ*_2_)
Corporate Fin.	0.92425	0.05026	0.0000	0.02549	0.84559	0.15329	0.00000	0.00112
SMEs Fin.	0.82172	0.16907	0.0000	0.00921	0.94289	0.02982	0.00000	0.02729
Agriculture Fin.	0.93038	0.0524	0.0000	0.01722	0.79796	0.19813	0.00000	0.00391
Consumer Fin.	0.79893	0.19314	0.0000	0.00793	0.95151	0.04398	0.00000	0.00451
Commodity Fin.	0.85719	0.10992	0.0000	0.03289	0.87591	0.09284	0.00000	0.03125
Other sectors Fin.	0.75487	0.23676	0.0000	0.00837	0.75794	0.22615	0.00000	0.01591
Total financing	0.86573	0.11236	0.0000	0.02191	0.8593	0.13171	0.00000	0.00899

* The decay factors 1 *-* (*δ*1 + *δ* 2) = 0.0162, where *δ* 1 = 0.9776, and *δ*2 = 0.0062; Max. Log-Likelihood = -19,239.2

** The decay factors 1 *-* (*δ* 1 + *δ* 2) = 0.0207, where *δ* 1 = 0.9728, and *δ* 2 = 0.0065; Max. Log-Likelihood = *-*19,628.7

Table 7 reports the maximum likelihood estimates of the correlation decay parameters (*δ*_1_ and *δ*_2_) and the volatility decay parameters (*λ*_1_ and *λ*_2_) for both variant of the DCC-GARCH. The results suggest that the decay parameters are highly significant for both models. The sum of the volatility decay parameters (*λ*_1_ and *λ*_2_) are less than 1 for each series which indicates that conditional volatilities are mean reverting and the volatility tend to decay gradually. Similarly, the sum of correlation decay parameters (*δ*_1_ and *δ*_2_) is also less than 1 for each time series which indicates the presence of mean reversion in the conditional correlations. This indicates that the system has tendency to return gradually to the normality. Thus, both variants of the DCC-GARCH model suggest that interest rate volatility has a relationship with the volatility of other financing portfolios and this relationship is mean reverting.

Consequently, benchmarking interest rate may lead to several implications for the financing (assets side) of Islamic banks, particularly in the dual banking system [34]. For example: First, due to the strong linkage between the two systems, arbitrage between the systems is possible, especially by customers who are indifferent towards both banking systems. This in turn implies that rates within the Islamic banking system must change with changes in the interest rate in the conventional system. Otherwise, rate differentials will prevail leading to arbitrage opportunities [60].

Secondly, due to opportunities of such risk free arbitrage through fund flows, Islamic banks become vulnerable to the consequences of interest rate movements that normally apply to conventional banks. For example, when the cost of funds changes in the conventional banking system, the costs of funds to Islamic banks will also change. Thus, bank may end up paying higher return to the depositors in the wake of avoiding the outflow of existing deposits and attracting new deposits. Consequently, the bank’s income margin may shrink due to increase in cost of funds on the liability side if the earnings from the assets do not keep pace with the interest rate movements. Thus, though the effect of interest rate variation may be indirect on the Islamic banking industry, the consequences would be similar [33, 34].

#### The unconditional volatilities and correlations

Table 8 reports the pairwise conditional correlations of interest rates with different streams of Islamic financing respectively. The correlations are ordered to highlight the ranking of different streams of Islamic financing in terms of their co-movement with the interest rate.

**Table 8:** Conditional correlations of interest rate with Islamic financing.

Rank	Financing Stream	Unconditional Volatility
1	Corporate Financing	1.3498
2	SMEs Financing	1.2992
3	Consumer Financing	1.2975
4	Agriculture Financing	1.2231
5	Commodity Financing	1.1024
6	Other sectors Financing	0.9559

The results indicate that interest rate has the highest correlation with the corporate financing (1.3498) and the lowest correlation with other sector financing (0.9559). This indicates that corporate financing, which constitute more than 70% of the overall financing portfolio of Islamic banking industry in Pakistan, is more vulnerable to interest rate risk followed by the SMEs financing. This is because the financial managers usually opt for an optimal capital structure, determined by the cost of financing and a trade-off between tax shield benefits and bankruptcy costs associated to debt financing including the financing from banks [98]. Thus, a change in interest rate triggers changes in cost of Islamic financing which ultimately leads to changes in the demand for Islamic financing. Thus, Islamic banks, though operating on interest free ideologies, are prone to interest rate risk.

Compared to other sectors commodity financing is relatively less affected by interest rate volatility. This is because commodity financing is predominantly composed of financing extended to government departments to support the prices of agriculture commodities via commodity operations. Whereby, its financing decision is not contingent upon the cost of financing or its volatility. Rather, the commodity operations are part of the government policy to support and sustain the prices of commodities in the market. Furthermore, government departments do not choose between Islamic and conventional banks on the basis of the relative difference in their cost of financing. Rather the government subsidiaries, upon the recommendations of finance division and SBP, acquire a portion of funds for commodity operations from Islamic banks to solve the excess liquidity problem being faced by Islamic banking industry in Pakistan. This is because, being in its nascence, the assets creation of Islamic banking industry is weak which induces liquidity management problems particularly in the short run [19].

## Discussion and implications

The results of Cointegration test suggest a significant long-term relationship while the VECM suggest short-term relationship between the interest rate volatility and the financing of Islamic banking industry. Similarly, the results of DCC-GARCH model also confirm the relationship between interest rate volatility and the volatility of financing portfolios of Islamic banking industry. These results invariably confirm the presence of strong links between the Islamic and the conventional banking systems due to benchmarking interest rate. Consequently, Islamic banks are prone to adverse effects of the interest rate movements.

These findings confirm the view of Archer and Karim [25], Bacha [34], and Rosly [30] who suggest that benchmarking interest rate exposes Islamic banks to the interest rate risk. In addition, these findings are not unique to Pakistan only. Rather, these results can be generalized for all countries that use the interest based benchmark rate. For example, Ito [78] investigates the association among the Islamic rate of return in Malaysia and conventional interest rates in the Malaysian deposit market and the KLIBOR (Kuala Lumpur Interbank Offered Rate) rates. His findings suggest a co-movement in the Islamic rate of return and interest rates in the Malaysian deposit market as well as the short term KLIBOR rates.

Even the Islamic banking industries of the GCC countries, despite using the Islamic Interbank Rate (IIBR) for price reference, are also prone to the interest rate risk. This is because the IIBR too is closely associated with the conventional benchmark rates [26, 31, 33, 44–46]. For example, Nechi and Smaoui [46] investigates a dynamic long run association between IIBR and conventional benchmark rate in the GCC countries including Kuwait, Bahrain, Qatar, UAE, and Saudi Arabia. Their findings suggest that a significant long run relationship between the IIBR and the respective conventional benchmark rates of these countries except Kuwwait and Qatar. Similarly, Fakhfekh, Hachicha [73] investigated the volatility dynamics of the Islamic and conventional banks in the GCC countries including Kuwait, Bahrain, Qatar, UAE, and Saudi Arabia. Their findings suggest that though Islamic banks operating the GCC countries are somehow more resilient than their conventional counterparts. But, they exhibit almost similar volatility patterns are also prone to the financial risks like conventional banks.

Furthermore these results are also consistent with the previous studies that suggest the adverse effect of benchmarking interest rate on the performance of Islamic banking in various ways [See for example 36, 38–40, 60, 64–69, 72]. These findings imply that Islamic banks have to confront several challenges in the current regulatory frameworks adopted by several central banks, primarily due to exposure to interest rates risk. First, in the dual banking systems, the implementation of interest rate changes in the context of monetary policy severely affects the Islamic banks both on assets and liability sides by exposing Islamic banks to the so-called rate of return risk [99]. The rate of return risk “stems from uncertainty in the returns earned by IBs on their assets when an increase in benchmark rates results in expectations of higher rates of return on investment” [100].

In fact, unlike conventional banks that can transfer the burden of interest rate rise to their borrowers in most cases (depending on their terms of lending) due to their basic business model of borrowing and lending, Islamic banks have very limited ability to do so. This is because, the modes of financing used by Islamic banks on the assets side either involve sale on credit, diminishing *Musharakah*, or leasing. In case of credit sale (e.g., *Murabahah*), the return is determined in advance and is included in the pre agreed selling price which cannot be changed subsequently in response to variation in the market cost of capital. The diminishing *Musharakah* and lease contracts, on the other hand, may allow periodic adjustments in the lease rentals based on an agreed benchmark. However, the adjustment period is too long (typically three months or even longer) to allow the Islamic banks to respond to the changes in the market cost of funds. Consequently, conventional banks, being able to lend at floating rates and also having recourse to interest rate swaps, are clearly at an advantageous position compared to Islamic banks.

This inability to adjust promptly to the changes in the benchmark rate makes Islamic banks vulnerable to the so-called "withdrawal risk". Since, Islamic banks typically use the partnership (or *Wakalah*) based fund management model on the deposit side, where Islamic bank acts as a managing partner while depositors act as the sleeping partners. This model allows the depositors to withdraw funds at short notice. Given this model, the asset-liability management because a challenge for Islamic banks, particularly when the benchmark rate rises, due to their inability to reflect this rise in their return on investment in the short run. Consequently, Islamic banks, not being able to offer a comparable return that could meet the market expectation of depositors, get exposed to the withdrawal risk and the resultant financial distress.

This may in turn expose Islamic banks to a third type of risk called the “displaced commercial risk” (DCR) which refers to the transfer of risk associated with deposits to the shareholders of the bank. This risk arises when the bank has to forgo a part of their profit, under commercial pressure, to pay the depositors in the wake of preventing withdrawals due to a lower return on deposits [101]. Thus, the benchmark rate risk if not managed properly, could pose a significant threat to the capital base, financing, and earnings of Islamic banks. Furthermore, variation in the interest rate could: i) make Islamic banks vulnerable to contingent risks form the off-balance sheet exposures, ii) affect its cost of capital, and iii) affect the underlying value of the assets of Islamic banks [For details see Archer and Karim (2019)]. Thus if these issues are not curtailed by the central banks, particularly in the regions where Islamic banks have a significant share in the overall banking assets, it would induce systematic risk and financial instability in the market.

## Conclusions and recommendation

The present study empirically examines the dynamic linkage between the interest rate volatility and the financing of Islamic banking industry. The findings suggest that a significant long-term and short-term relationship exists between the interest rate volatility and the financing of Islamic banking industry providing significant evidence for co-movements and convergence. The study therefore concludes that invariably strong links exist between the Islamic and the conventional banking systems due to benchmarking interest rate. Therefore, Islamic banks, though operating on interest free ideologies, are prone to interest rate risk (IRR) which ultimately induces the rate of return risk, withdrawal risk, and displaced commercial risk in Islamic banking.

Thus, the benchmark rate risk, if not managed properly, could pose a significant threat to the capital base, financing, and earnings of Islamic banks. Moreover, it can induce systematic risk and financial instability in the market. Therefore, the central banks should introduce and allow *Shariah* compliant model contracts for Islamic finance that allow for periodic adjustments of lease rentals or profit rate. This would help the banks in dealing with the adverse effects of monetary policy, particularly the exposure to rate of return risk, which would consequently lead to financial stability. Furthermore, based on the findings it is strongly recommended that Islamic banks should have their own unique *Shariah* compliant non-interest based benchmark that is significantly decoupled from the conventional benchmarks and could be used as a robust indicator of the average expected cost of short term interbank market funding. This would not only help in establishing a unique identity of Islamic banks but would also provide a cushion against potential losses resulting from probable future financial crises. Furthermore, it would help in resolving the issue of Islamic banks’ exposure to the interest rate risk, rate of return risk, and withdrawal risk.

Efficient local non-interest based benchmark rates are also desirable for the Islamic banking industries coexisting in the dual banking systems like Pakistan keeping in view the changing dynamics in the international and local financial environment whereby, the LIBOR has finally been phased out starting 2022, while its counterpart Euribor is also losing its attraction. Similarly, their alternative benchmark rate like SONIA, SARON and SOFR are already being experimented with. Similarly, the prevailing Islamic interbank benchmark rate (IIBR) has also failed to prove as independent reference rates due to its strong linkage with the conventional benchmark rates. This calls for the focused and serious coordination among the digitization experts, regulators, practitioners and academia to come up with more innovative and workable solutions. However, to have such a benchmark the Islamic banking industry has to offer unique and distinctive financial products having unique risk-return profiles. If Islamic banks continue to compete with the financial products of conventional banks by mimicking their products, the Islamic banks would remain a sub-set of the conventional banks with similar products and rates.

## Supporting information

S1 Data (XLSX)
